# Muscle glycome in idiopathic inflammatory myopathies: Impact in IL-6 production and disease prognosis

**DOI:** 10.1016/j.isci.2023.107172

**Published:** 2023-06-17

**Authors:** Ana Campar, Inês Alves, Beatriz Santos-Pereira, Rafaela Nogueira, Miguel Mendonça Pinto, Carlos Vasconcelos, Salomé S. Pinho

**Affiliations:** 1 Institute for Research and Innovation in Health (i3S), University of Porto, Porto, Portugal; 2 Institute of Biomedical Sciences Abel Salazar (ICBAS), University of Porto, Porto, Portugal; 3 Clinical Immunology Unit, Porto University Hospital Centre, Porto, Portugal; 4 Faculty of Medicine, University of Porto, Porto, Portugal; 5 Department of Chemistry, NOVA School of Science and Technology, Lisbon, Portugal; 6 Neuropathology Department, Porto University Hospital Centre, Porto, Portugal

**Keywords:** Health sciences, Disease, Glycobiology, Glycomics

## Abstract

Idiopathic inflammatory myopathies (IIM) are a group of chronic autoimmune diseases mainly affecting proximal muscles. Absence of meaningful prognostic factors in IIM has hindered new therapies development. Glycans are essential molecules that regulate immunological tolerance and consequently the onset of autoreactive immune response.

We showed that muscle biopsies from patients with IIM revealed a deficiency in the glycosylation pathway resulting in loss of branched *N*-glycans. At diagnosis, this glycosignature predicted disease relapse and treatment refractoriness. Peripheral CD4^+^ T cells from active-disease patients shown a deficiency in branched *N*-glycans, linked to increased IL-6 production. Glycan supplementation, restoring homeostatic glycosylation profile, led to a decrease in IL-6 levels.

This study highlights the biological and clinical importance of glycosylation in IIM immunopathogenesis, providing a potential mechanism for IL-6 production. This pinpoints muscle glycome as promising biomarker for personalized follow-up and a potential target for new therapies in a patients’ subgroup with an ominous evolution.

## Highlights

• A specific tissue glycosignature in patients with IIM predicts poor disease course
• Loss of branched *N*-glycans in peripheral T cells associates with IL-6 production in IIM
• *Ex vivo* glycan supplementation of IIM biopsies results in decreased IL-6 levels
• Muscle glycome acts as a prognostic biomarker and a potential therapeutic target

## Graphical abstract

**Figure:**
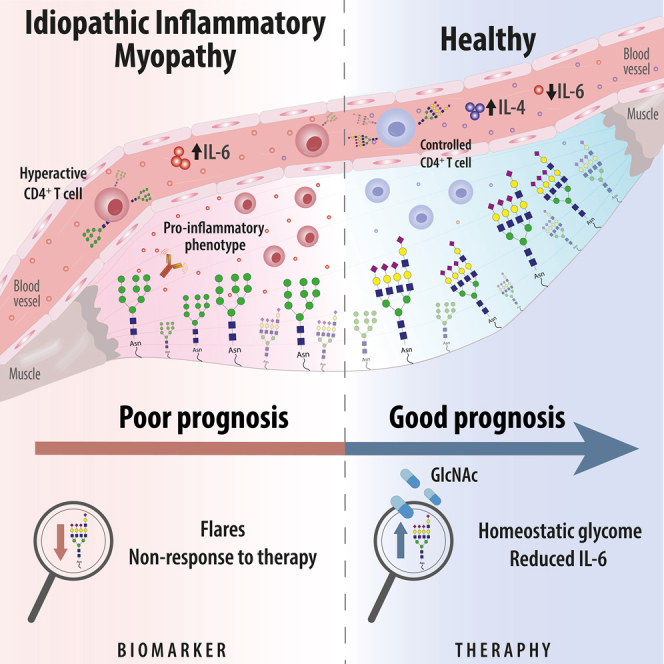

## Introduction

Immune-mediated or idiopathic inflammatory myopathies (IIM) are a group of rare systemic autoimmune diseases characterized by proximal skeletal muscle weakness leading to long-term disability, decreased quality of life, and reduced life expectancy. IIM are generally characterized by an increase in muscle enzymes, with or without skin involvement. In addition, other organs may also be affected in a large proportion of patients, namely the gastrointestinal tract, lungs, and heart, often resulting in a poor prognosis.^1^ Notwithstanding recent advances in the classification of the disease,^2^ clinically the most important subtypes of IIM are dermatomyositis, namely the subgroup of amyopathic dermatomyositis, inclusion body myositis, immune-mediated necrotizing myopathy, and overlap myositis (which includes the subgroup of anti-synthetase syndrome). Polymyositis appears to be an increasingly dying entity.^3^

The etiology of IIM is still unknown, but a relationship among environmental triggers, genetic risk factors, and defective immunoregulatory mechanisms appears to play an important role in the pathogenesis of the disease, particularly with an important, albeit unexplored, role for the interleukin (IL)-6 cytokine.^4^^,^^5^ IIM share many features with other systemic autoimmune diseases such as rheumatoid arthritis and systemic lupus erythematosus. It is a chronic, lifelong, and recurrent disease that primarily affects occupationally active people and is associated with significant morbidity, economic burden, and mortality.^6^^,^^7^ Although survival in these patients has improved since the widespread use of corticosteroids and immunosuppressants, mortality remains elevated in patients with IIM. The 5-year survival rate has been reported to be 60%, especially in the first year after diagnosis.^8^ The increased morbidity is primarily due to severe muscle weakness and visceral involvement. Recent reports suggest that only 20%–40% of treated patients achieve remission, while 60%–80% experience a polycyclic or chronic continuous disease progression, which has a major impact on quality of life at mid- and long-term follow-up, as up to 80% of treated patients remain disabled.^6^

One of the notable features of IIM is the presence of myositis-specific antibodies, which have been used to predict the manifestations of IIM. At our center, we have found that the presence of Ro52 antibodies and the overlap with other autoimmune diseases appear to be independent variables associated with the development of damage and refractoriness in IIM and act as predictors of the worst prognosis (data not published). However, there has long been debate about the extent to which the presence of the various autoantibodies commonly associated with IIM may represent accurate biomarkers for the detection of IIM subcategories or behave as epiphenomena of the disease process.^9^ On the other hand, about 15%–20% of patients are seronegative, i.e. they do not display significant levels of autoantibodies. Therefore, there is an urgent need in the clinical setting for accurate and reliable biomarkers to improve the classification and diagnosis of IIM subgroups and to obtain significant reliable prognostic information about disease progression, as this lack of diagnostic and prognostic biomarkers hinders major advances in the treatment of these diseases.^10^^,^^11^^,^^12^

Furthermore, the therapeutic tools available for the treatment of IIM are limited. The choice of different immunotherapeutic drugs is mainly empirical, and standard treatment guidelines are not conservative and not disease/subgrouping-directed. The reasons for this include the rarity of IIM, its heterogeneous clinical phenotype and lack of consensual classification criteria, as well as the small number of randomized, double-blind controlled clinical trials.^13^

Given these major problems in the clinical and therapeutic management of IIM and the significant medical and societal impact of the disease, there is an urgent need in clinics for the identification and characterization of key molecular parameters/biomarkers that could contribute to the correct diagnosis of the disease subgroup at an early stage of disease progression and, more importantly, to the stratification of patients at increased risk of developing aggressive disease and premature mortality. This knowledge will certainly improve the course of the disease and the rate of therapeutic success, paving the way for the development of novel targeted therapeutic strategies that will ultimately have a positive impact on patient survival and quality of life.

Glycosylation is a major post-translational modification that occurs in essentially all cells. It is characterized by the enzymatic attachment of glycans (sugar chains) to other saccharides, proteins, and lipids, resulting in a diverse and abundant repertoire of glycans on the cell surface collectively known as glycome. Glycans have important biological functions within a cell^14^ and are extremely important as master regulators of the immune system. Changes in cellular glycome, including immune cell glycome, can occur in response to environmental and genetic stimuli and are often associated with the acquisition of altered cellular phenotypes such as malignancy and chronic inflammation.^14^^,^^15^^,^^16^^,^^17^

In particular, we and others have demonstrated the importance of glycans as regulators of various immunological mechanisms, including adaptive immunity, especially involving T cells.^18^^,^^19^ Glycans have been reported to control the rate of T cell receptor (TCR) endocytosis and modulate cellular responses through interaction with lectins (such as galectins) and their ligands.^20^ A reduction in the branching of *N*-glycans (in the context of a malfunction/deficiency of *N*-acetlyglycosaminil transferase V (GnT-V) glycosyltransferase) on T cells has been shown to lead to an increased clustering of TCRs and consequently a lower threshold for T cell activation.^16^ This was demonstrated by our group in patients with ulcerative colitis, in whom a deficiency of branched glycans on intestinal T cells was associated with T cell hyperactivity and increased disease severity.^19^ In addition, GnT-V-deficient mice were shown to have an enhanced delayed-type hypersensitivity response and increased susceptibility to experimental autoimmune encephalitis (EAE)^18^ and inflammatory bowel disease (IBD).^21^ Treatment of these mice with high concentrations of *N*-acetylglucosamine (GlcNAc) increased GnT-V-mediated *N*-glycan branching and inhibited TCR activation and autoimmune responses in mouse models of EAE, IBD, and type 1 diabetes. Furthermore, there is evidence for a role of glycosylation in immune cell differentiation, e.g. in the generation and phenotypic profiles of T helper (Th) cell subsets, particularly activated CD4^+^ effector T cells (which differentiate into TH1, TH2, TH17, and regulatory T cells), in part through its effect on the production of ligands of various lectins. In particular, *N*-glycan branching has been shown to play an important role in autoimmunity by promoting the differentiation of anti-inflammatory TH2 cells over pro-inflammatory TH17 cells.^22^

In addition to the role of glycans as direct regulators of immune cell function, we have also shown that alterations in epithelial cell glycosylation have an impact on the loss of self-tolerance. We have shown that impairment of *N*-glycan branching at the renal cell surface contributes to the pathogenesis of lupus and serves as a potential prognostic biomarker in lupus nephritis.^23^

In the present work, we aimed to characterize the clinical and biological impact of muscle glycome in the immunopathogenesis of IIM, in particular how glycosylation changes at peripheral immune cells regulate the systemic immune response and IIM prognosis. We demonstrated for the first time a clear truncation of the branching *N*-glycosylation pathway in muscle tissue of patients with IIM, which was associated with a poor prognosis. Importantly, we observed that this glycosylation alteration could be detected in the periphery, where circulating CD4^+^ T cells exhibit deficient *N*-glycosylation associated with IL-6 production. This pathogenic immunophenotype was reversed by metabolic supplementation with glycans from fresh muscle biopsies.

This study contributes to the pathophysiological knowledge of IIM, focusing on epigenetic alterations that may contribute to the pathogenesis evolution and prognosis of this group of rare and heterogeneous diseases. Targeted and more effective therapy relies on a deeper knowledge of the pathogenesis implied in etiology, evolution, and expected prognosis.

## Results

### IIM muscle biopsies display an altered *N*-glycosylation profile associated with poor disease course and non-response to therapy

**Figure 1:**
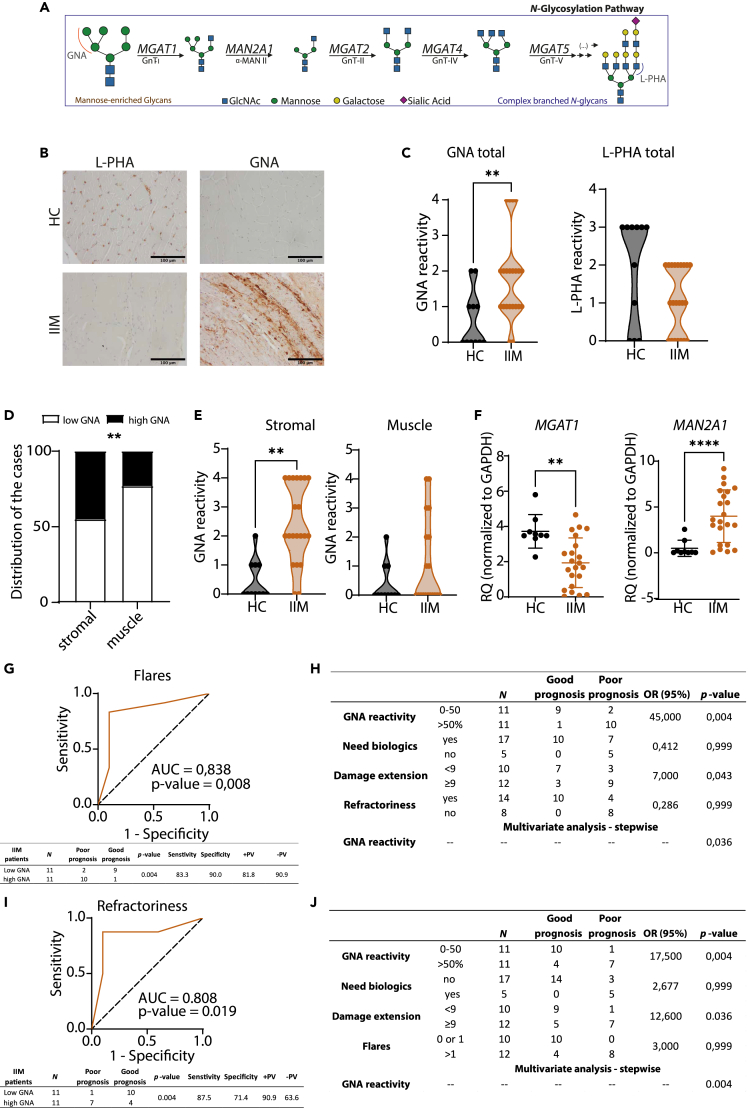
Altered *N*-glycosylation profile of muscle biopsies at diagnosis associates with disease course and response to therapy in patients with IIM (A) Schematic representation of the *N*-glycosylation pathway from high-mannose *N*-glycans (recognized by GNA lectin) toward more complex *N*-glycans, with β1,6-branching antennae (detected by L-PHA lectin). (B) Representative images of skeletal muscle from IIM and HC, low L-PHA, and high GNA reactivity in IIM biopsies (scale bar = 100 μm). (C) Qualitative score of GNA and L-PHA staining intensity from total tissue from IIM and HC biopsies. Analysis performed for 22 IIM and 11 HC cases. Unpaired one-tailed Mann-Whitney tests (∗p value<0.05, ∗∗p value<0.01). (D) Distribution of IIM disease cases in high (≥50%) and low (<50%) GNA reactivity, at each cellular component (muscular or stromal). Unpaired one-tailed Mann-Whitney tests (∗p value<0.05, ∗∗p value<0.01). (E) Quantitative analysis of staining intensity in each cellular compartment (muscular or stromal) of IIM compared with HC. Unpaired one-tailed Mann-Whitney tests (∗p value<0.05, ∗∗p value<0.01). (F) *MGAT1* and *MAN2A1* mRNA expression levels on total muscle FFPE biopsies from patients with IIM and HC individuals. Each dot represents one biological sample. Relative quantification (RQ) of mRNA levels is expressed as mean ± SD (Mann-Whitney t-test: ∗p value<0.05, ∗∗p value<0.01). (G) ROC curve for the GNA reactivity from patients with IIM with poor disease course (more than 1 flare) and its respective AUC and p value. (H) The predictive capacity of high GNA reactivity and other clinic-pathological parameters to distinguish poor disease prognosis (that have more than 1 flare). (I) GNA reactivity ROC curve, respective AUC and p value, for IIM patients’ refractory to treatment. (J) The predictive capacity of high GNA reactivity and other clinic-pathological parameters to distinguish non-responder patients (that do not respond to therapy). The error bars represent the standard deviation of the data.

Taking in consideration the natural progression of *N*-glycans from high-mannose precursors toward more complex and branched structures (Figure 1A), we aimed to obtain a simple glycosignature from the IIM skeletal muscle based on the levels of these two distant *N*-glycan traits. To do so, histochemistry of GNA and L-PHA lectins were used, to assess the relative levels of high-mannose and β1,6-branched *N*-glycans (respectively) in a cohort of 22 IIM muscle tissue (IIM) and 11 healthy muscle tissue (HC). The demographic and clinical features of patients with IIM are included in the Table S1. The results showed an overall increased reactivity of GNA in muscle biopsies from patients with IIM, compared with HC, together with a decreased L-PHA staining (Figure 1B), despite not statistically significant for L-PHA (Figure 1C). Interestingly, we observed that the major GNA reactivity was detected in the stromal component of the tissue, with less reactivity in the muscle fibers (Figures 1D and 1E). This tissue glycoprofile was further confirmed at transcriptional level, by determining the expression of glycogenes involved in the *N*-glycosylation pathway, such as *MGAT1* and *MAN2A1* (Figure 1A). IIM biopsy cells seem to be significantly deficient in the expression of *MGAT1* (Figure 1F), a gene that encodes for *N*-acetylglucosaminyltransferase-I (GnT-I), a key enzyme that adds the first GlcNAc antenna, converting high-mannose into hybrid *N*-glycan structure. Surprisingly, *MAN2A1*, the gene that encodes for α-mannosidase (an enzyme that acts immediately into the GnT-I product) was increased (Figure 1F), which may be explained by a compensatory mechanism to recover the progression in the *N*-glycosylation pathway and maintain the homeostatic glycan repertoire.

Given our previous knowledge that alterations in the tissues glycome might be associated with pathogenesis and prognosis of the disease,^23^^,^^24^ we explored, at a longitudinal level, the predictive capacity of GNA reactivity at diagnosis to the disease outcome. We have observed that high GNA reactivity in the stromal compartment (staining for more than 50% of the cells) at diagnosis was able to stratify patients according to the risk of having more than 1 flare (poor prognosis), with a specificity of 90% and sensitivity of 83.3% (Figure 1G). The univariate analysis also demonstrated that high GNA reactivity at diagnosis increased 45-times the odds (p value = 0.004) of patients with IIM to have more than 1 flare at 3 years of follow-up. Interestingly, multivariate analysis revealed that among other clinical parameters used to monitor the disease progression, high GNA reactivity detected at muscle biopsy was the only variable that predicts the development of more than 1 flare in an independent way (Figure 1H). Moreover, high GNA reactivity was also able to identify refractory patients (defined as those that do not respond to 2 immunosuppressive drugs given, concomitantly or subsequently, for at least 3 months) with a specificity of 71.4% and sensitivity of 87.5% (Figure 1I). The univariate analysis showed that high GNA reactivity at diagnosis increased 18-times the odds (p value = 0.004) of patients with IIM to be non-responders at 3 years of follow-up. Multivariate analysis revealed that among other clinical parameters used to monitor the disease, high GNA reactivity at muscle biopsy was the only independent variable that predicts the non-response to treatment (Figure 1J).

### Deficiency in complex type *N*-glycans at the surface of peripheral immune cells is associated with pro-inflammatory systemic profile in patients with IIM

**Figure 2:**
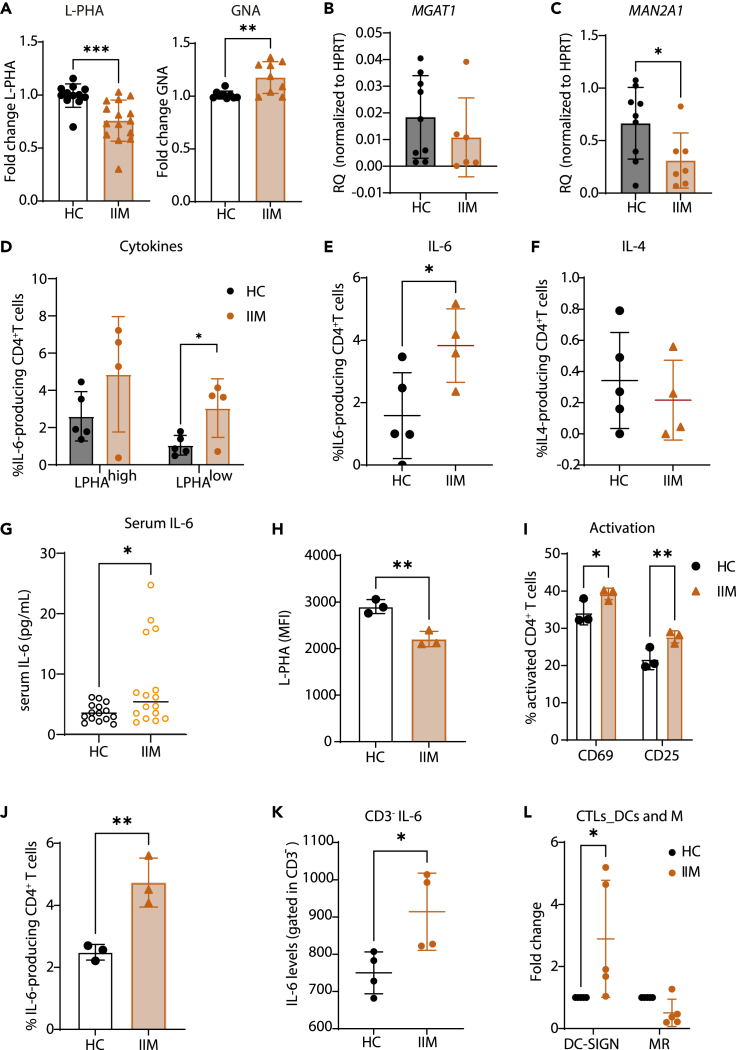
Altered *N*-glycosylation profile of CD4+ T cells associates with increased activation and cytokine production in patients with IIM (A) Cell surface β1,6-branching *N*-glycans (L-PHA) and high-mannose (GNA) *N*-glycans of CD4^+^ T cells from IIM and HC peripheral blood, analyzed by flow cytometry. Levels of median fluorescence intensity (MFI) from patients with IIM were normalized for the average of healthy control MFI. Fold change ratio is represented as mean ± SD. Unpaired one-tailed Mann-Whitney tests (∗p value<0.05, ∗∗p value<0.01). (B) *MGAT1* and (C) *MAN2A1* mRNA expression levels on peripheral CD3^+^ T cells from patients with IIM and HC individuals. Each dot represents one biological sample. Relative quantification (RQ) of mRNA levels is expressed as mean ± SD (Mann-Whitney t-test: ∗p value<0.05,∗∗p value<0.01). (D) Percentage of IL-6-producing CD4^+^ T cells in peripheral blood, gated on CD4^+^ T cells with high vs. low levels of L-PHA staining, from patients with IIM and HC individuals. Percentage of cells are expressed as mean ± SD. Unpaired one-tailed Mann-Whitney test (∗p value<0.05, ∗∗p value<0.01). (E) Levels of total IL-6-producing CD4^+^ T cells, analyzed by flow cytometry. Percentage of cells are expressed as mean ± SD. Unpaired one-tailed Mann-Whitney tests (∗p value<0.05, ∗∗p value<0.01). (F) Levels of total IL-4-producing CD4^+^ T cells, analyzed by flow cytometry. Percentage of cells are expressed as mean ± SD. Unpaired one-tailed Mann-Whitney tests. (G) Serum IL-6 quantification by ELISA from IIM and HC. Levels are expressed as mean ± SD. Unpaired one-tailed Mann-Whitney test (∗p value<0.05, ∗∗p value<0.01). (H) Cell surface β1,6-branching *N*-glycans (L-PHA) of CD4^+^ T cells from IIM and HC peripheral blood after 24 h stimulated with 0.5 μg/mL anti-CD3, analyzed by flow cytometry. Levels are expressed as mean ± SD. Unpaired one-tailed Mann-Whitney tests (∗p value<0.05, ∗∗p value<0.01). (I) Percentage of activated (CD25 or CD69) CD4^+^ T cells from IIM and HC peripheral blood after 24 h stimulated with 0.5 μg/mL anti-CD3, analyzed by flow cytometry. Percentages are expressed as mean ± SD. Unpaired one-tailed Mann-Whitney test (∗p value<0.05, ∗∗p value<0.01). (J) Percentage of IL-6-producing CD4^+^ T cells from IIM and HC peripheral blood after 24 h stimulated with 0.5 μg/mL anti-CD3, analyzed by flow cytometry. Percentages are expressed as mean ± SD. Unpaired one-tailed Mann-Whitney test (∗p value<0.05, ∗∗p value<0.01). (K) Levels of IL-6 produced by CD3^−^ cells, analyzed by flow cytometry. Percentages are expressed as mean ± SD. Unpaired one-tailed Mann-Whitney tests (∗p value<0.05, ∗∗p value<0.01). (L) Median fluorescence intensity (MFI) of DC-SIGN and mannose receptor (MR) in DCs and macrophages from IIM and HC. Levels are expressed as mean ± SD. Unpaired one-tailed Mann-Whitney test (∗p value<0.05, ∗∗p value<0.01). The error bars represent the standard deviation of the data.

Taking in consideration that stromal glycoprofile has shown to be more significantly affected with glycosylation alterations (Figure 1E), we further analyzed the glycoprofile of immune cell population at the periphery, exploring a possible less invasive *proxy* of the *in situ* glycobiomarker. We have found that CD4^+^ T cells display significantly lower levels of surface β1,6-branched *N*-glycans, concomitantly with significantly increased levels of high-mannose *N*-glycans, detected by L-PHA and GNA reactivity, respectively (Figures 2A and S1A). The transcriptional profile of isolated peripheral CD3^+^ T cells showed no differences in *MGAT1* gene expression (Figure 2B), but revealed a deficient expression of the gene *MAN2A1*, which may result in the lower hydrolysis of one of the mannose branches precluding the addition of a second GlcNAc antennae and thus leading to the accumulation of less complex *N*-glycans (Figure 2C). Given the previous evidence showing that loss of β1,6-branched *N*-glycans in CD4^+^ T cells has an impact on the TCR signaling and T cell function,^16^^,^^18^^,^^21^ we analyzed the impact of this glycosylation alteration in CD4^+^ T cell-producing cytokines, namely IL-6 (one of the key cytokines in the etiopathogenesis of IIM). To do so, we stratified CD4^+^ T cells into L-PHA high vs. L-PHA low. Interestingly, we have observed that L-PHA low subpopulation showed a significant increase in these IL-6-producing cells in IIM (Figures 2D and S1B). Overall, and as expected, CD4^+^ T cells from patients with IIM are more prone to produce IL-6, but no differences were found for other cytokines, such as IL-4 (Figures 2E and 2F). Accordingly, serum IL-6 levels from our cohort were shown to be significantly increased in active patients with IIM compared to controls (Figure 2G). Moreover, and to show the dependency of TCR glycosylation in defining the threshold for activation, we have cultured IIM and HC peripheral blood mononuclear cells in the presence of anti-CD3 antibody and checked for the activation and cytokine production of CD4^+^ T cells. As expected, CD4^+^ T cells from patients with IIM, displaying lower levels of β1,6-branched *N*-glycans (Figure 2H), revealed increased levels of TCR-dependent activation (Figure 2I) and increased IL-6 production (Figure 2J).

Furthermore, we also observed that non-T cells (CD3^−^) from patients with IIM may also contribute for the elevated levels of IL-6 in these patients (Figure 2K), which may include dendritic cells and monocytes, among others. Interestingly, dendritic cells from patients with IIM seem to express higher levels of DC-SIGN, a glycan receptor that recognizes high-mannose *N*-glycans (Figure 2L), implicating a role for innate immunity in the hyperactive environment of IIM.

### *Ex vivo* glycosylation reprograming of muscle tissue with glycans supplementation recovers branching *N*-glycans composition and hampers IL-6 production *in situ*

**Figure 3:**
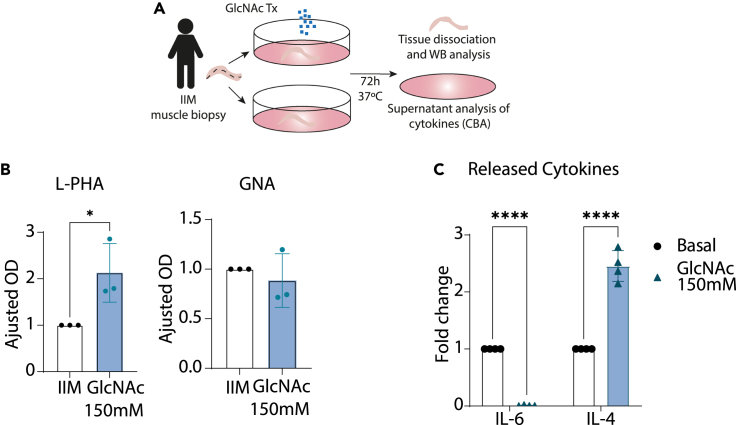
GlcNAc supplementation of fresh muscle biopsies from IIM patients attenuates pro-inflammatory immune response (A) Experimental outline of 150 mM *N*-acetylglucosamine (GlcNAc) supplementation of IIM biopsies for 72 h. Each patient biopsy was longitudinally cut into 2 similar portions, one for the supplementation (GlcNAc 150 mM) and other for control without supplementation (IIM). After incubation, cells were lysed for protein extraction and lectin blot analysis, together supernatant which was collected and released cytokines were analyzed by cytokine bead assay (CBA). (B) Adjusted values of intensity from L-PHA and GNA lectin blot of whole tissue cell lysate with or without GlcNAc supplementation. Normalized for loading control (actin). Levels are expressed as mean ± SD. Mann-Whitney tests (∗p value<0.05, ∗∗p value<0.01). (C) Fold change of cytokines secreted to the supernatant by IIM biopsies supplemented with 150 mM GlcNAc for 72 h, normalized by the respective basal condition. Levels of released cytokine were analyzed by cytokine bead assay (CBA) One-way ANOVA followed by Tukey’s post test.∗p < 0.05, ∗∗p < 0.01, ∗∗∗p < 0.001. Levels are expressed as mean ± SD.

In order to repair the deficiency in the *N*-glycosylation pathway observed in Figure 1, we have promoted the progression toward branching *N*-glycosylation and thus restoring the levels of branching *N*-glycans. Fresh muscle biopsies from patients with IIM were collected and supplemented *ex vivo* with GlcNAc (Figure 3A). This increases the substrate availability to GnTs and consequently promote the expression of β1,6-branching *N*-glycans on tissue, as previously done by us.^21^^,^^25^ We have observed a significant increase in the overall levels of β1,6-branching *N*-glycans in tissue glycoproteins (Figures 3B and S2A) which was not significant for high-mannose *N*-glycans (Figures 3C and S2A). We showed that in fact, muscle T cells are a target of glycosylation modification, such as complex branched *N*-glycans (Figures S2B and S2C). Importantly, the increase in β1,6-branching *N*-glycans resulted in a significant reduction of IL-6 production together with an increase of IL-4 cytokine (Figure 3C).

## Discussion

Muscle cells surface is enriched in glycans and glycoproteins (the glycocalyx) which role in muscle homeostasis and function remains largely unexplored. In fact, changes in glycosylation are a hallmark among many diseases including cancer, chronic inflammation, and autoimmune diseases.^15^^,^^17^^,^^23^^,^^26^ However, whether and how the muscle glycome has a clinical and biological impact in the immunopathogenesis of IIM remains completely unknown. Here, we demonstrated that in fact muscle biopsies from patients with IIM exhibit an abnormal glycosylation profile characterized by a decreased expression of complex branching *N*-glycans along with an increased expression of mannose-enriched glycoproteins, in comparison with normal controls. These alterations in glycosylation signature appear to be, predominantly observed, in the stroma compartment and inflammatory infiltrate. However, and in order to precisely analyze the structural glycosylation profile of infiltrating muscle T cells, mass spectrometry analysis should be performed in future allowing a better structural insight of the muscle glycome and correlation with the pathogenesis of muscle diseases. Importantly from the clinical standpoint, we showed that this specific glycosignature was found to have prognostic value in patients with IIM. We demonstrated that levels of high-mannose *N*-glycan content in stroma of muscle biopsies, detected at diagnosis, are correlated with a poor disease course (more flares) and non-response to therapy. High GNA reactivity in muscle biopsy, at diagnosis, proved to be an independent variable in predicting the development of more than 1 flare, as well as in identification of refractory patients, with a good sensitivity and specificity, when compared with currently available tools/markers. These tissue-specific glycosylation alterations have been previously reported in other autoimmune diseases, namely in kidney from lupus nephritis,^23^^,^^25^ synovial fibroblasts from rheumatoid arthritis,^27^ as well as fucosylation in IBDs^28^ and sialylation in GNE myopathy.^29^

At the periphery, our findings in IIM revealed that CD4^+^ T cells from peripheral blood of patients with IIM display the same truncation of the *N*-glycosylation pathway as observed in the tissue, suggesting that CD4^+^ T glycoprofile can be a proxy for what is happening at the muscle tissue, potentially constituting a promising non-invasive biomarker in IIM. Interestingly, we found that peripheral CD4^+^ T cells with the lower levels of L-PHA (from patients with IIM) seem to present lower TCR thresholds, with increased activation markers and IL-6 production. Accordingly, in 2009, Okayama and colleagues published an elegant study refereeing to the critical role of IL-6 in a murine model of myositis.^5^ They found increased IL-6 mRNA levels in the mice muscles (particularly in macrophages) which correlated with the histological severity of myositis. IL-6 was proved to be essential for the development of myositis, in this model, and its blockage suppressed the incidence and severity of myositis. No further studies developing this mechanism were published yet, but IL-6 blockade has been effectively used in some patients with IIM.^30^^,^^31^ Further studies, using larger cohorts, are needed to understand if this peripheral CD4^+^ T cell-specific glycosignature may be associated with clinical variables that allow a better monitorization of the patients with less invasive techniques.

The origin for this glycan alteration in the immune compartment of affected muscle in patients with IIM still needs further investigation. However, some mechanisms have been proposed by others and us to explain the link between glycosylation changes and T cells hyperactivity and a pro-inflammatory immune response.^16^ In fact, β1,6-branched *N*-glycans were shown to be essential regulator of TCR thresholds in CD4^+^ T cells in both IBD^19^ and multiple sclerosis.^18^ In IBD, we showed that a deficiency in branched *N*-glycans in mucosa T cells from patients with ulcerative colitis was associated with disease severity and T cell hyperactivity.^21^

Additionally, we also found that innate immune cells from patients with IIM display an increased capacity to recognize tissue glycosylation alterations as we have observed an increased expression of DC-SIGN in innate immune cells that specifically recognize mannose structures. This observation may underlie the impact of muscle glycosylation alteration in innate immune recognition and consequent potentiation of the pro-inflammatory immune response, as we have observed in lupus,^25^ an issue that deserves further investigation.

Therapeutic strategies in immune-mediated systemic diseases, historically and presently, rely on immunosuppression, to reduce the hyperreactivity underlying clinical symptoms and organ dysfunction. Side effects, particularly infections (the main cause of flares in IIM), are the dark face of this approach. Theoretically, restoring immune homeostasis is much more appealing and a more natural way to treat the fundamental problem of these patients, but much harder to achieve. Based in our findings, we propose the glycosylation reprogramming of the muscle as an attractive strategy to recover a homeostatic glycome toward the natural progression of branching *N*-glycosylation with consequences in immune homeostasis by hampering IL-6 production. Overall, glycosylation remodeling by metabolic supplementation with glycans may constitute an appealing therapeutic strategy to attenuate the pro-inflammatory immune response in IIM and might represent a novel target for directed therapy, eventually associated with immunosuppression, in a subset of patients with a hazardous evolution and altered glycosignature.

### Limitations of the study

Despite the promising impact of this study in identifying a mechanism that underlies IIM immunopathogenesis, one of the limitations is the retrospective nature of the analysis of the clinical parameters defining poor prognosis. A prospective cohort to validate the findings will be worth. The number of muscle samples from controls was low (comparing to patients), although enough for statistical analysis. The limited sample size of patients with IIM and subgroups precluded some statistical significance supporting the need to validate the mechanism in a larger and prospective cohort of IIM samples and controls.

## STAR★Methods

### Key resources table

**Table:** 

REAGENT or RESOURCE	SOURCE	IDENTIFIER
**Antibodies**		

FVD APC-eFluor780	eBioscience	Cat#65-0865-14
L-PHA-fluorescein, FITC	Vector Laboratories	Cat#FL-1111
GNA biotinylated	Vector Laboratories	Cat#B-1245
Streptavidin PE-Cy7	eBioscience	Cat#25-4317-82; RRID:AB_10116480
BV421 anti-human CD14 (clone 63D3)	Biolegend	Cat#367144; RRID:AB_2810580
PE-Cy7 anti-human CD206 (clone 15-2)	Biolegend	Cat#321123; RRID:AB_10900995
eFluorTM 450 anti-human CD4 (clone RPA-T4)	eBioscience	Cat#48-0049-41; RRID:AB_1272127
APC anti-human CD11c (clone BU15)	eBioscience	Cat#17-0128-42; RRID:AB_11151141
PE anti-human TCRα/β (clone BW242/412)	Miltenyi	Cat#130-098-887; RRID:AB_2661264
BV510 anti-human CD3 (clone OKT3)	BD Biosciences	Cat#69-0037-42; RRID: AB_2848508
Polyclonal DC-SIGN rabbit IgG	Biorad	Cat#AHP627; RRID:AB_323683
Polyclonal swine anti-rabbit IgG (FITC)	DAKO	Cat#E0354
Pacific Blue IL-6 (clone MQ2-13A5)	Biolegend	Cat# 501113; RRID: AB_2561425
APC IL-4 (clone MP4-25D2)	Biolegend	Cat# 500811; RRID: AB_315130
L-PHA biotinylated	Vector Laboratories	Cat#B-1115
Mouse IgG anti-actin (C4)	Santa Cruz	Cat#SC-47778; RRID: AB_626632
anti-CD4 antibody [EPR6855]	Abcam	Cat# ab133616; RRID: AB_2750883
GNA-fluorescein	Vector Laboratories	Cat# FL-1241-2
PE Donkey anti-rabbit IgG	Biolegnd	Cat#406421 RRID: AB_2563484
Anti-human TCRβ polyclonal antibody	Santa Cruz Biotechnology	Cat#sc-9101 RRID: AB_661720

**Biological samples**		

FFPE Muscle biopsies	Neuropathology Department of Centro Hospitalar Universitário do Porto	https://www.chporto.pt/v0C0B0G0K/neuroporto
Human PBMCs	Clinical Immunology Unit, Porto University Hospital Centre, Porto, Portugal	https://www.chporto.pt/v0C0B0E0M/unidade-multidisciplinar-de-imunologia-clinica

**Chemicals, peptides, and recombinant proteins**		

Lymphoprep^TM^	Stemcell Technologies	Cat#07801
FBS Premium Heat Inactivated	VWR	Cat#S181H
Mouse serum	Jackson Immunoresearch	Cat# 015-000-120
Phorbol 12-myristate 13-acetate, PMA	Sigma	Cat#P8139
Ionomycin	Sigma	Cat#I0634
Brefeldin A	Sigma	Cat#B7651
Xylene	Fisher Chemical	Cat#1330-20-7
Alcohol 99%	Enzymatic	Cat#64-17-5
30% Hydrogen Peroxide	Fisher Chemical	Cat#7722-84-1
3,3'-diaminobenzidine, DAB	Sigma	Cat#D4293
Haematoxylin	Epredia	Cat#720804
Entellan® new	Sigma	Cat#107961
SuperScript IV Reverse Transcriptase	Invitrogen	Cat# 18090050
Random hexamer primers	Invitrogen	Cat#48190-011
RPMI 1640 Medium, GlutaMAX^TM^ supplement, HEPES	Gibco^TM^	Cat#72400-054
Penicillin-Streptomycin (10000 U/mL)	Gibco^TM^	Cat#15140122
N-Acetyl-D-glucosamine, GlcNAc	Sigma	Cat#A8625
Trizma® base	Sigma	Cat#T6066
Hydrochloric Acid, HCl	Merck	Cat#109057
NaCl	Sigma	Cat#S9888
EDTA	Sigma	Cat#E5134
Nonidet P-40	Sigma	Cat#I3021
PMSF	Roche	Cat#10837091001
Sodium ortovanadate	Sigma	Cat#S6508
Complete Protease Inhibitor Cocktail	Roche	Cat#50-100-3301
TMB (3,3',5,5'-tetramethylbenzidine) chromogen	Invitrogen	Cat#88-7314-88
2N H_2_SO_4_	Merck	Cat#100731
10% bis-acrylamide	Serva	Cat#10688
0.45 μm nitrocellulose membranes	Amersham	Cat#10600002
Tween-20	Sigma	Cat#1379
Bovine Serum Albumin, BSA	Sigma	Cat#A7906
ECL	Amersham	Cat#RPN2106
Rabbit serum	DAKO	Cat#X0902

**Critical commercial assays**		

Foxp3/Transcription factor staining buffer Set	eBioscience	Cat#00-5523-00
Vectastain ABC kit	Vector Laboratories	Cat#PK-6100
RecoverAll™ Total Nucleic Acid Isolation Kit	Invitrogen	Cat#AM1975
Mouse ELISA Ready-SET-Go! Kits	eBioscience	Cat#88706488
BD Cytometric Bead Array Human Th1/Th2/Th17 Kit	BD Bioscience	Cat# 551811
Protein G Sepharose™ 4 Fast Flow	Cytiva	Cat#17061801

**Oligonucleotides**		

*MAN2A1-FAM* TaqMan probe	Applied Biosystems	Cat# Hs01123597
*MGAT1-FAM* TaqMan probe	Applied Biosystems	Cat# Hs00159121
*GAPDH-FAM* TaqMan probe	Applied Biosystems	Cat# Hs02758991

**Software and algorithms**		

BD Diva	Becton Dickinson	N/A
FlowJo v10	Becton Dickinson (Tree Star Inc)	N/A
Prism 9	GraphPad software	N/A
Image Lab Software	BIO-RAD	N/A
Statistical software SPSS v25	IBM Corp	N/A

### Resource availability

#### Lead contact

Further information and requests for resources and reagents should be directed to and will be fulfilled by the lead contact, Salomé S. Pinho (salomep@ipatimup.pt).

#### Materials availability

This study did not generate new unique reagents.

### Experimental model and study participant details

To characterise the glycoprofile of human skeletal muscle in IIM, 22 formalin-fixed and paraffin-embedded (FFPE) muscle biopsies from IIM patients were selected from the neuropathology department of the Centro Hospitalar Universitário do Santo António (CHUSA) for lectin histochemistry and gene expression analysis. The study population reflects the nature of this group of diseases, which themselves are rare and heterogeneous. Muscle biopsies were selected taking into account availability of samples (as not all patients with suspected myositis undergo muscle biopsy for various reasons), origin of samples (more recent samples were preferred due to their better preservation) and quality of samples (definitive histologic myositis changes were preferred over non-specific changes or samples with low quantity or poor tissue quality) (Table S1). Blood samples were collected from patients who currently had active disease, regardless of treatment.

In addition, 11 FFPE muscle biopsies from healthy controls were used. All samples were collected from patients undergoing a scheduled muscle biopsy (2019–2021) at CHUSA. Biopsies were performed at diagnosis without treatment, all were surgical biopsies, and analyses were performed in 2 steps: fresh samples for immunohistochemistry and haematoxylin-eosin, along with genetic analyses, in preserved samples for each and all patients.

All participants gave informed consent and the procedures were approved by the CHUSA Ethics Committee (2017-155 (132- DEFI 124-CES)).

Poor prognosis was classified as patients who were refractory (defined as patients who did not respond to 2 concurrent or subsequent immunosuppressive drugs for at least 3 months^32^ or required biologic treatment, or > had 1 relapse in the last 4 years.

#### Human PBMCs isolation

Human peripheral blood mononuclear cells (PBMCs) from IIM patients and healthy donors were isolated by gradient centrifugation using LymphoprepTM (Stemcell Technologies). The upper phase, containing the serum, was collected and stored at −80°C. PBMCs (interphase) were collected, washed twice with PBS and incubated with fixable viability dye (FVD)-APC-eFluor780 (eBioscience) for 30 min. Cells were washed with PBS and resuspended in FACS buffer (PBS 2%FBS).

#### Flow cytometry staining

For lectin staining, cells were incubated with conjugated lectins (Vector Laboratories): Phaseolus Vulgaris leucoagglutinin (L-PHA fluorescein, FITC), Galanthus Nivalis lectin (GNA-biotinylated) for 15 min. Subsequently, non-specific binding was prevented by blocking with 2% mouse serum in FACS for 10 min and the biotinylated lectin was incubated with streptavidin-PE-Cy7 (eBioscience) for 30 min. For surface marker staining, cells were stained for 30 min on ice and protected from light with the following antibodies: BV421 anti-human CD14 (clone 63D3), PE -Cy7 anti-human CD206 (clone 15-2) from Biolegend; eFluorTM 450 anti-human CD4 (clone RPA-T4), APC anti-human CD11c (clone BU15), from eBioscience; PE anti-human TCRα/β (clone BW242/412) from Miltenyi; BV510 anti-human CD3 (clone OKT3) from BD Biosciences; for DC-SIGN staining, cells were incubated with polyclonal DC-SIGN rabbit IgG (Biorad), followed by incubation with polyclonal porcine anti-rabbit IgG (FITC; Dako) for 30 min on ice. Cells were resuspended in FACS buffer prior to analysis. For intracellular staining, cells were previously incubated with 200 ng/mL phorbol myristate acetate (PMA, Sigma), 20 ng/mL ionomycin (Merck) and 100 ng/mL Brefeldin A (Sigma) for 5 h. Fixation and permeabilization was performed using the Foxp3/transcription factor staining buffer set (eBioscience) according to the manufacturer’s instructions. Finally, cells were incubated with Pacific Blue IL -6 (MQ2-13A5, Biolegend) and APC IL-4 (MP4-25D2, Biolegend) for 30 min. Data were obtained using a BD FACS Canto II instrument (Becton Dickinson) and analysed using FlowJo v10.0 (Tree Star Inc.).

#### Lectin histochemistry

FFPE muscle tissue sections (3 μm) from IIM patients (n=22) and healthy controls (n=11) were analysed by lectin histochemistry. For glycan profile determination, sections were deparaffinised, rehydrated and endogenous peroxidase activity blocked (3% H_2_O_2_). Sections were then incubated with the biotinylated Phaseolus Vulgaris Leucoagglutinin (L-PHA) lectin, which recognises β1,6GlcNAc-branched *N*-glycans, or with the biotinylated Galanthus Nivalis agglutinin (GNA) lectin, which is used for the detection of terminal α-1,3 mannose residues. The avidin-biotin-peroxidase complex was detected using the Vectastain ABC kit and the colour was developed using 3,3'-diaminobenzidine (DAB, Thermo Scientific, DE, USA). L-PHA, GNA and the Vectastain ABC kit were obtained from Vector Laboratories, Burlingame, CA, USA. Finally, all sections were counterstained with haematoxylin, dehydrated and preserved in appropriate embedding medium (Entellan® new, Merck Millipore).

#### Immunofluorescence staining

FFPE muscle tissue sections (3 μm) were double stained for CD4 receptor and high-mannose (GNA). Firstly, sections were deparaffinized and rehydrated, followed by antigen retrieval in citrate buffer for 40 min. After blocking with rabbit serum (x0902, DAKO) in BSA 10% (1:5) for 30 min, samples were incubated with anti-CD4 antibody (1:500, EPR6855, Abcam) and GNA-FITC conjugated lectin (1:100, Vector Laboratories) for 2 h in the dark. Then, the samples were washed 3 times for 10 min with PBST 0.01%, followed by incubation with donkey anti-rabbit PE-conjugated secondary antibody (ref 406421, Biolegend) in BSA 10% (1:200) for 1 h. Lastly, cell nuclei were stained with DAPI in PBS (1:100) for 5 min at RT and images were recorded in Zeiss Axio Imager Z1.

#### mRNA expression

Total RNA was isolated from 20 μm sections of formalin-fixed, paraffin-embedded (FFPE) tissue samples from muscle IIM patients (n=22) using the RecoverAll™ Total Nucleic Acid Isolation Kit (Invitrogen) according to the manufacturer's protocol. Total RNA was quantified using the Nanodrop 1000 system and 100 μg was transcribed into single-stranded cDNA using SuperScript II Reverse Transcriptase and Random Hexamer Primers (Invitrogen, Oregon, USA) according to the manufacturer's recommendations. Quantitative real-time PCR (qRT-PCR) was performed in duplicate for the following target genes: *MAN2A1*-FAM (Hs01123597) and *MGAT1*-FAM (Hs00159121), both TaqMan probes from Applied Biosystems, and the housekeeping gene *GAPDH*-FAM (Hs02758991). Reactions were performed using a 7500 Fast Real-time PCR System (Applied Biosystems™) and mRNA data were analysed using the comparative 2(-ΔΔCT) method, normalised to housekeeping gene mRNA expression.

#### Human IIM muscle explant supplementation

A fragment of each IIM muscle biopsy was cut longitudinally into two similar sections. One of the sections was incubated in 200 μL of complete RPMI supplemented with 150 mM GlcNAc at 37°C and 5% CO_2_ for 72 h. The other part of the biopsy was incubated in the same medium. The other section of the biopsy was incubated under the same conditions but without the addition of GlcNAc. At the end of the incubation period, the supernatants were collected for cytokine analysis and the biopsy parts were weighed. Protein lysates were obtained by dissociating the tissue with a pestle in lysis buffer (50 mM TrisHCl, pH 7.4, 150 mM NaCl, 1 mM EDTA, 1% Nonidet P-40) supplemented with protease and phosphatase inhibitors, 100 mm PMSF, 100 mM Sodium ortovanadate and Complete Protease Inhibitor Cocktail (Roche).

#### ELISA and CBA

IL-6 concentration in sera from IIM and healthy donors (mouse ELISA Ready- SET -Go! kits from eBioscience) was measured by ELISA according to the manufacturer’s protocol. TMB (3,3',5,5'-tetramethylbenzidine) chromogen solution (eBioscience) was used as substrate and 2N H_2_SO_4_ as stop solution. Absorbance was measured at 450 nm and 570 nm using a microplate reader (Biotek Instruments).

For the supplemented human muscle biopsies, cytokine concentrations were analysed by flow cytometry using cytometric bead arrays: the BD Cytometric Bead Array Human Th1/Th2/Th17 Kit (BD Bioscience), according to the manufacturer's instructions. Samples were measured on the BD Accuri C6 instrument (BD Biosciences, US) using a specific template provided by BD Biosciences.

#### Lectin blot

For analysis of high mannose compared to complex *N*-glycans on the surface of the IIM biopsy cell population, SDS-PAGE -dissolved proteins (10% bis-acrylamide) were transferred to 0.45 μm nitrocellulose membranes (Amersham) and incubated in blocking solution (PBS 0.05% Tween-20 and 5% BSA) ON at 4°C. Membranes were then incubated with 3.5 μg/mL biotinylated L-PHA for 1 h at room temperature, washed 3 times with PBS 0.05% Tween 20 and incubated with ECL (Amersham) for 30 min prior to development with the Vectastain ABC kit. Mouse IgG anti-actin (C4, Santa Cruz) was used for loading control analysis.

#### Immunoprecipitation and Western-blot

For the immunoprecipitation (IP) of the TCRαβ, protein was obtained from total cell lysates of muscle biopsies treated with GlcNAc or non-treated from IIM patients. Equal amounts (30 μg of protein) of protein were precleared 60 min, at 4°C with Protein G Sepharose™ 4 Fast Flow beads (Cytiva, Sweden) and the supernatant was incubated with polyclonal rabbit anti-human TCRβ antibody (H-197; Santa Cruz Biotechnology) overnight, at 4°C. The immune complex was incubated with Protein G-Sepharose beads, for 2 h, at 4°C under rotation. Then, it was boiled at 96.5°C for 5 min to release the immune complex. The samples were separated by 10% SDS–PAGE electrophoresis and transferred onto nitrocellulose membranes (Amersham). For the TCRβ, the membrane was blocked with 5% non-fat milk and then probed with the primary anti-human TCRβ polyclonal antibody (1:100) and revealed with secondary antibody goat anti-rabbit IgG-HRP (1:2000; GE Healthcare, Life Sciences). For the β1,6 GlcNAc branched glycans, the membrane was blocked with 4% Bovine Serum Albumin (BSA, Sigma) and then incubated with the biotinylated L-PHA (10 μg/mL) lectin and revealed with the VECTASTAIN® ABC kit peroxidase (Vector Labs, Burlingame, CA, USA) kit. The detection was performed by the ECL reagent (Amersham). Quantitative analyses were performed by densitometric scanning of bands (GS-900 Calibrated Densitometer) and analyzed in Image Lab software (BIO-RAD).

#### *In vitro* TCR stimulation

Human peripheral blood mononuclear cells were isolated as stated above. After washing, 200 000 cells were cultured for 24h, at 37 ⁰C in 96-well round bottom plates coated with anti-CD3 monoclonal antibody (mAb clone OKT3, 0.5 μg/mL eBioscience™) in 200 μL of Roswell Park Memorial Institute (RPMI) medium supplemented with 10% fetal bovine serum (FBS), 1% Penicillin-streptomycin (Pen-Strep). For the analysis of TCR-dependent IL-6 production, 100 ng/mL of Brefaldin A (Sigma) were added to the wells.

### Quantification and statistical analysis

Data visualisation and statistical analyses (non-parametric Mann-Whitney t-test) were performed using GraphPad Prism 9 software.

The predictive power of GNA reactivity to discriminate patients with poor disease progression from those with good disease progression was determined by plotting receiver operating characteristic curves (ROC) and calculating the area under the curve (AUC). Adaptability was assessed using the Hosmer-Lemeshow statistic and test. Results are presented in the form of odds ratios (ORs) for each category compared to a predefined reference category and their respective 95% confidence intervals (CIs). Odds ratios above one or below one are indicative of a higher or lower probability, respectively, of developing a poor disease outcome compared to a reference category.

Datapoints were tested for outliers using ROUT testing and excluded when identified.

Statistical analysis was performed using Statistical Software version 25 (IBM Corp., IBM SPSS Statistics for Windows, version 25.0, Armonk, NY; published 2017). The threshold for statistical significance was p-value < 0.05.

## Data Availability

This study did not generate new datasets. This study does not report original code.
