# Supplementary material for: Muscle glycome in idiopathic inflammatory myopathies: Impact in IL-6 production and disease prognosis
Source: iScience. 2023 Jun 17;26(7):107172. doi: 10.1016/j.isci.2023.107172 (PMC10316658; doi:10.1016/j.isci.2023.107172)

iScience, Volume *26*

Supplemental information

Muscle glycome in idiopathic inflammatory myopathies: Impact in IL-6 production and disease prognosis

Ana Campar, Inês Alves, Beatriz Santos-Pereira, Rafaela Nogueira, Miguel Mendonça Pinto, Carlos Vasconcelos, and Salomé S. Pinho

Supplemental table 1: Demographic and clinical characteristics of patients with IIM (related to STAR methods).

Demographic characteristics		N (%)
Gender	19♀; 3♂	-
Age at diagnosis (mean)	48 years-old	-
Clinical characteristics		
Diagnosis	Dermatomyositis	10 (45.4)
	Polymyositis	3 (13.6)
	IMNM	3 (13.6)
	Anti-synthetase syndrome	3 (13.6)
	Inclusion Body Myositis	2 (9.1)
	Overlap myositis	1 (4.5)
ACR/EULAR criteria – IIM probability (mean)	86.3	-
Years of disease (Dec/ 2021) (mean)	6.4	-
Organic involvements	Muscle	22 (100)
	Skin	13 (59.1)
	Bulbar	13 (59.1)
	Lung	6 (27.3)
	Articular	6 (27.3)
	Other	6 (27.3)
Autoantibodies	Specific	17 (77.3)
	Associated	9 (40.9)
Refractoriness	Yes	8 (36.4)
Flares, last 4 years (mean)	2.5	-
Damage (MDI, mean)	MDI extension	9.14
	MDI severity	45.23
Treatments	Corticosteroids	21 (95.4)
	Methotrexate	9 (40.9)
	Azathioprine	10 (45.4)
	Mycophenolate mofetil	3 (13.6)
	Tacrolimus	4 (18.2)
	IV Immunoglobulin	15 (68.2)
	Cyclophosphamide	4 (18.2)

	JAK inhibitors<br>Biological (Rituximab)	1 (4.5)<br>4 (18.2)
Overlap with other AI diseases	Yes	6 (27.3)
Mortality	-	3 (13.6)
Prognosis	Good<br>Poor	10 (45.5)<br>12 (54.5)

IIM – Idiopathic Inflammatory Myopathy; IMNM - Immune Mediated Necrotizing Myopathy; MDI – Myositis Damage Index; IV – intravenous; JAK – Janus Kinase; AI – autoimmune.

Supplemental Figure 1 – Flow cytometric analysis (related to figure 2). (A) Gating Strategy of peripheral blood mononuclear cells analysis by flow cytometry. **(B)** Gating Strategy of peripheral blood mononuclear cells analysis for IL-6-producing CD4⁺T cells analysis by flow cytometry.

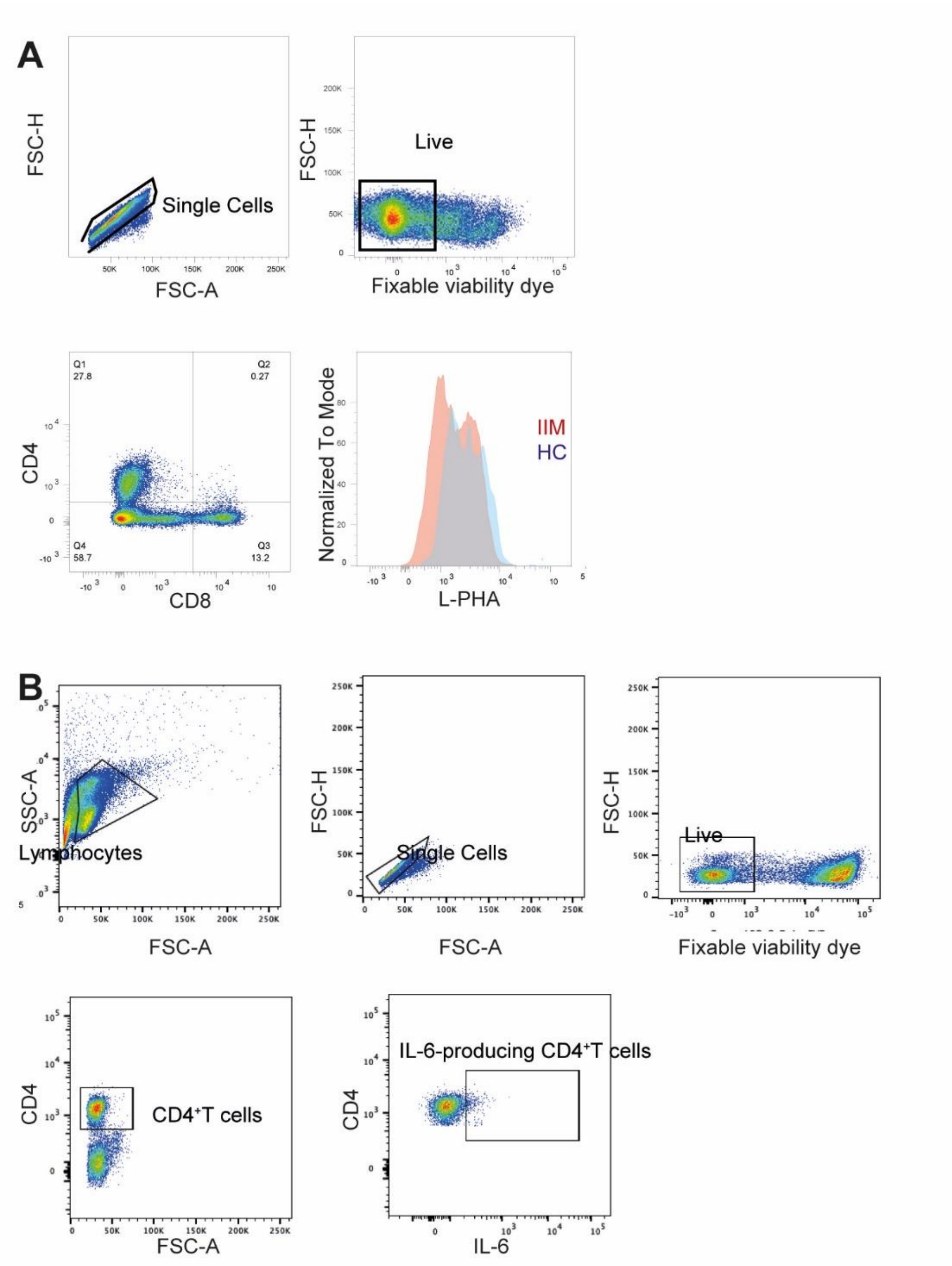

Supplemental Figure 2 – Glycoprofile of IIM muscle lesions (related to figure 3). (A) Lectin blot for L-PHA (β1,6-branhcing *N*-glycans) and GNA (high-mannose *N*-glycans) reactivity of whole tissue cell lysate with (Tx) or without (B) GlcNAc supplementation. Loading control (actin) of respective samples. **(B)** Immunofluorescence images representing the co-localization of CD4 (red) and L-PHA (green) at the stromal compartment of muscle biopsies from healthy controls (HC) and myositis (IIM). **(C)** Immunoprecipitation (IP) of TCRβ from IIM-derived muscle biopsies, treated and non-treated with GlcNAc. IP was stained with L-PHA lectin and anti-TCRβ antibody for the normalization of L-PHA reactivity. On the right is represented the quantification of the bands. Adjusted optical density (Adj. OD). The error bars represent the standard deviation of the data.

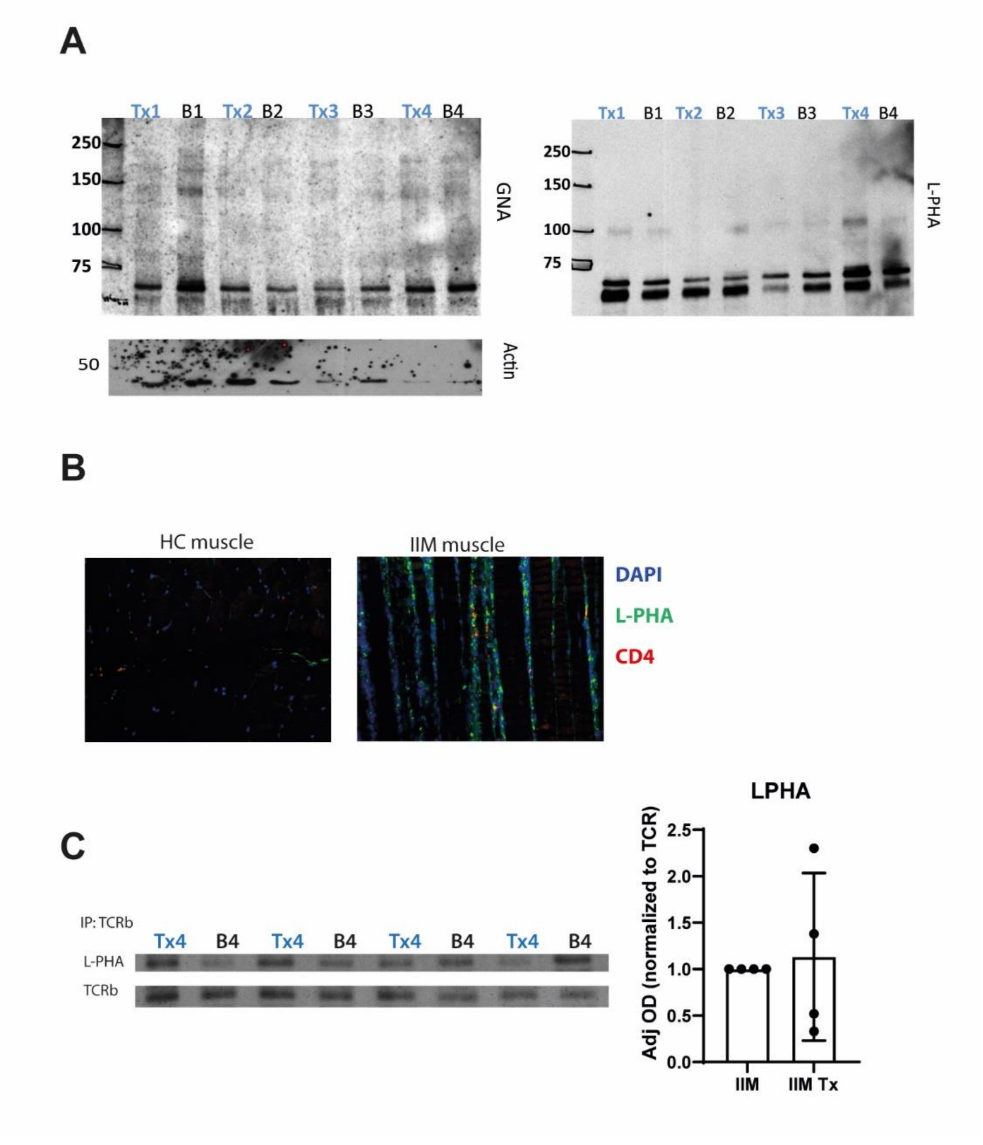

Supplement: Document S1. Figures S1 and S2 and Table S1 [file mmc1.pdf]
